# Supplementary material for: Cytoplasmic Localization of Thyroid Hormone Receptor (TR) Alpha and Nuclear Expression of Its Isoform TRα2 Determine Survival in Breast Cancer in Opposite Ways
Source: Cancers (Basel). 2023 Jul 13;15(14):3610. doi: 10.3390/cancers15143610 (PMC10377287; doi:10.3390/cancers15143610)
Supplement: Supplementary file 1 [file cancers-15-03610-s001.zip › cancers-2480347-supplementary.pdf]

Suppl. Figure S1: Control stainings for all antibodies used in this study

Positive control staining for TRα in thyroid tissue

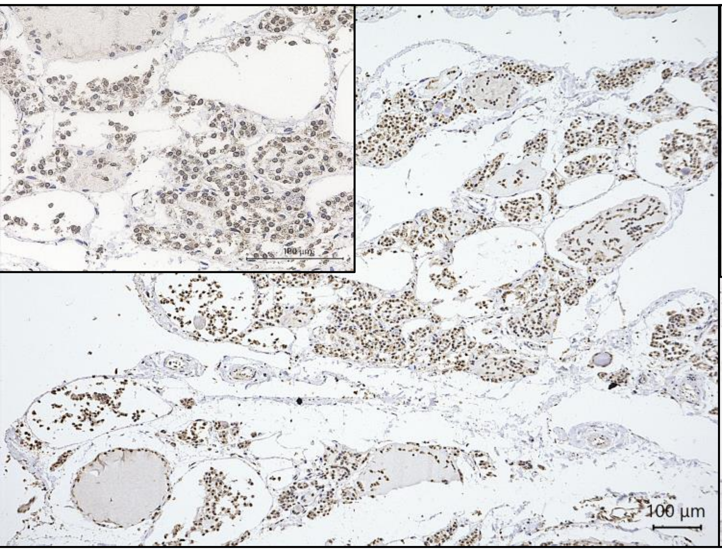

Negative control staining for TRα in thyroid tissue

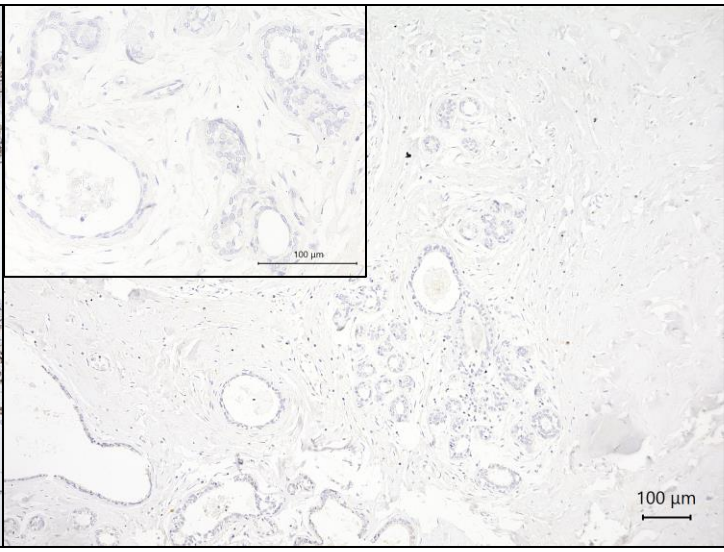

Positive control staining for TRα2 in human skin

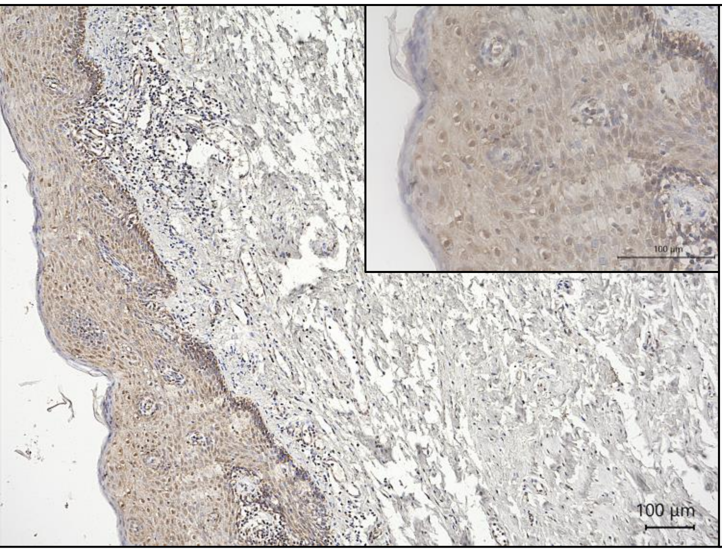

Negative control staining for TRα2 in human skin

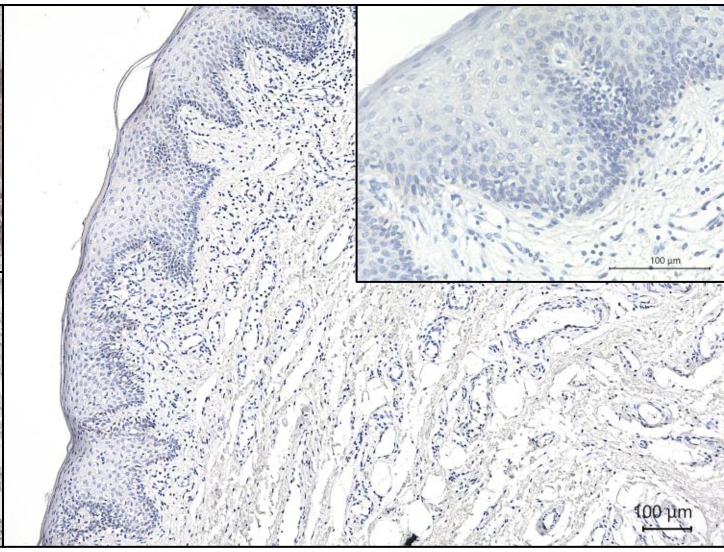

Positive control staining for TRα1 in human placenta and colon

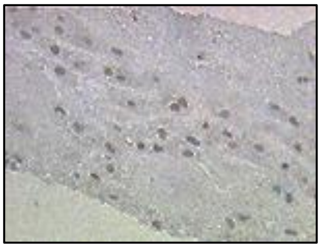

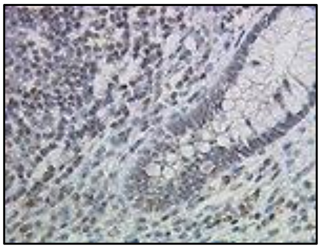

Negative control staining for TRα1 in human placenta

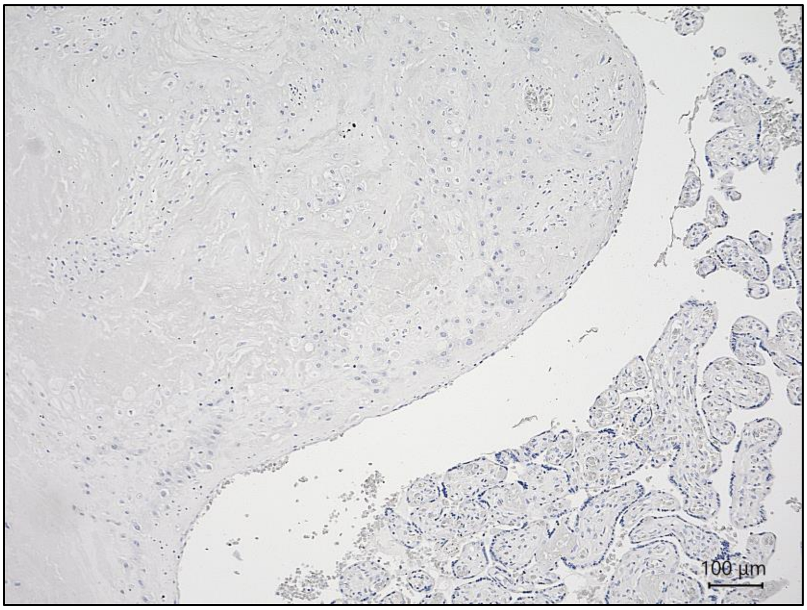

Suppl. Figure S2: Survival Function of nuclear TRα, TRα is not a significant prognosticator for overall Survival, ten year survival or disease free survival

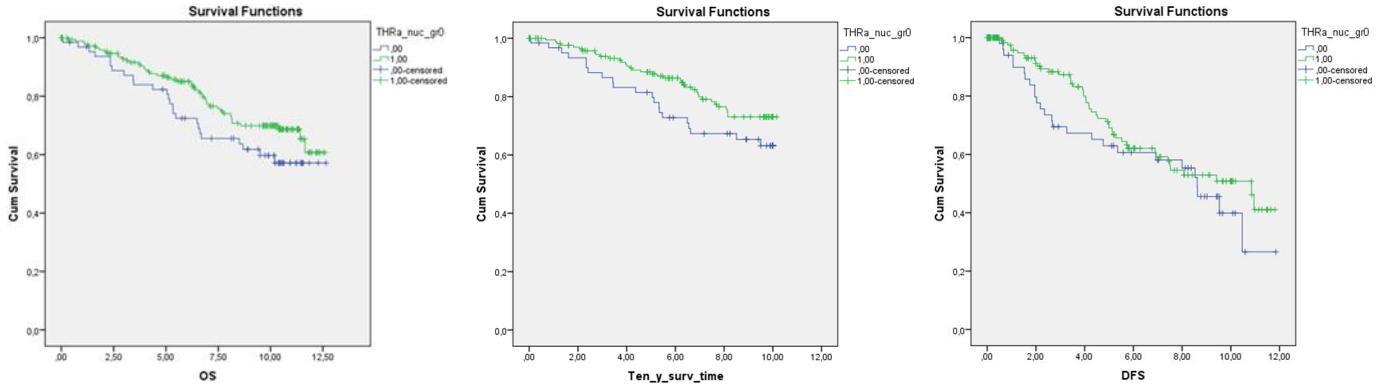

Means for Survival Time				
THRa_nuc_gr0	Mean			
	Estimate	Std. Error	95% Confidence Interval	
			Lower Bound	Upper Bound
0	9.39	0.537	8.337	10.442
1	10.246	0.299	9.66	10.833
Overall	10.026	0.265	9.506	10.546
a. Estimation is limited to the largest survival time if it is censored.				
Overall Comparisons				
	Chi-Square	df	Sig.	
Log Rank (Mantel-Cox)	2.34	1	0.126	
Test of equality of survival distributions for the different levels of THRa_nuc_gr0.				

Means for Survival Time				
THRa_nuc_gr0	Mean			
	Estimate	Std. Error	95% Confidence Interval	
			Lower Bound	Upper Bound
0	7.93	0.405	7.136	8.725
1	8.82	0.205	8.417	9.222
Overall	8.605	0.189	8.236	8.975
a. Estimation is limited to the largest survival time if it is censored.				
Overall Comparisons				
	Chi-Square	df	Sig.	
Log Rank (Mantel-Cox)	2.582	1	0.108	
Test of equality of survival distributions for the different levels of THRa_nuc_gr0.				

Means for Survival Time				
THRa_nuc_gr0	Mean			
	Estimate	Std. Error	95% Confidence Interval	
			Lower Bound	Upper Bound
0	7.213	0.637	5.965	8.462
1	8.129	0.406	7.333	8.926
Overall	7.872	0.345	7.197	8.548
a. Estimation is limited to the largest survival time if it is censored.				
Overall Comparisons				
	Chi-Square	df	Sig.	
Log Rank (Mantel-Cox)	1.395	1	0.238	
Test of equality of survival distributions for the different levels of THRa_nuc_gr0.				

Suppl. Figure S3: Survival Function of cytoplasmic TR$\alpha 2$, cytoplasmic TR$\alpha 2$ is not a significant prognosticator for overall Survival, ten year survival or disease free survival

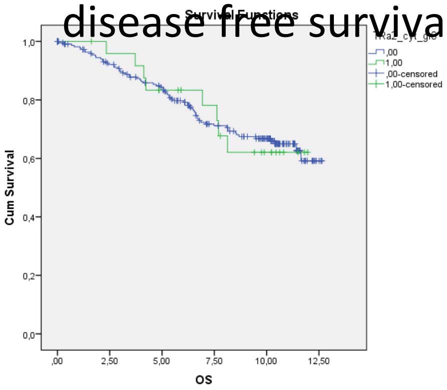

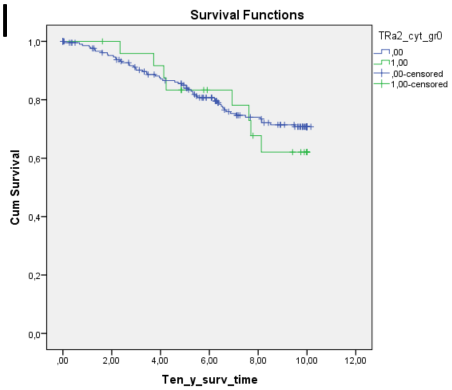

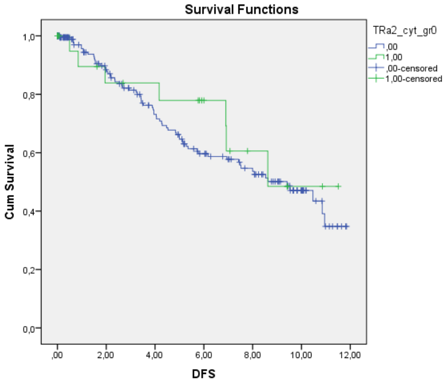

Means for Survival Time				
TRa2_cyt_gr0	Mean			
	Estimate	Std. Error	95% Confidence Interval	
			Lower Bound	Upper Bound
0	9.921	0.281	9.371	10,471
1	9.642	0.685	8.299	10,984
Overall	9.932	0.264	9,414	10,449
a Estimation is limited to the largest survival time if it is censored.				

Overall Comparisons			
	Chi-Square	df	Sig.
Log Rank (Mantel-Cox)	0,002	1	0,969
Test of equality of survival distributions for the different levels of TRa2_cyt_gr0.			

Means for Survival Time				
TRa2_cyt_gr0	Mean			
	Estimate	Std. Error	95% Confidence Interval	
			Lower Bound	Upper Bound
0	8,53	0,203	8,131	8,928
1	8,425	0,498	7,45	9,4
Overall	8,526	0,19	8,154	8,899
a Estimation is limited to the largest survival time if it is censored.				

Overall Comparisons			
	Chi-Square	df	Sig.
Log Rank (Mantel-Cox)	0,278	1	0,598
Test of equality of survival distributions for the different levels of TRa2_cyt_gr0.			

Means for Survival Time				
TRa2_cyt_gr0	Mean			
	Estimate	Std. Error	95% Confidence Interval	
			Lower Bound	Upper Bound
0	7,725	0,358	7,024	8,426
1	8,249	0,964	6,359	10,138
Overall	7,814	0,337	7,153	8,475
a Estimation is limited to the largest survival time if it is censored.				

Overall Comparisons			
	Chi-Square	df	Sig.
Log Rank (Mantel-Cox)	0,428	1	0,513
Test of equality of survival distributions for the different levels of TRa2_cyt_gr0.			

Suppl. Figure S4: Survival Function of cytoplasmic TRα1, cytoplasmic TRα1 is not a significant prognosticator for overall Survival, ten year survival or disease free survival

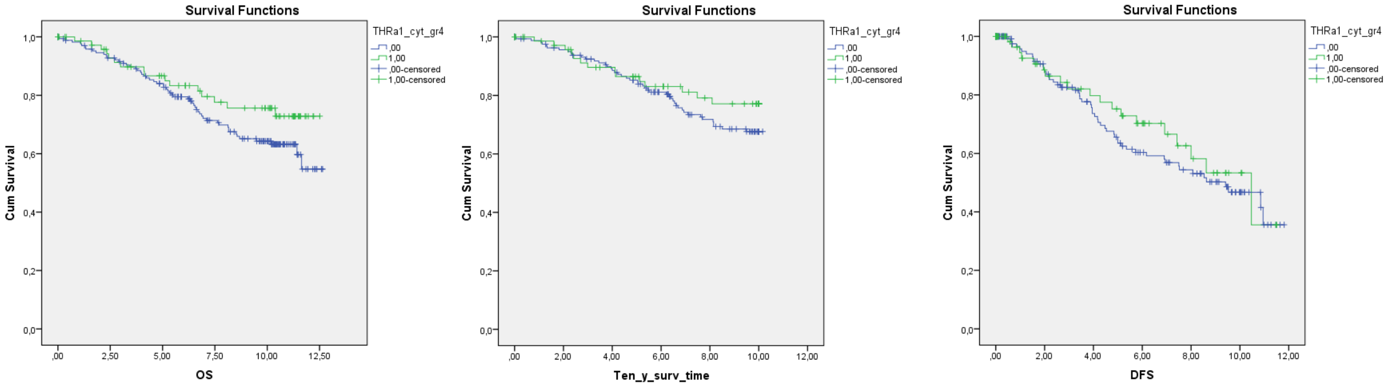

Means for Survival Time				
THRa1_cyt_gr4	Mean			
	Estimate	Std. Error	95% Confidence Interval	
			Lower Bound	Upper Bound
0	9,812	0,317	9,19	10,433
1	10,484	0,455	9,593	11,376
Overall	10,035	0,263	9,52	10,551

a Estimation is limited to the largest survival time if it is censored.

Overall Comparisons			
	Chi-Square	df	Sig.
Log Rank (Mantel-Cox)	2,053	1	0,152

Test of equality of survival distributions for the different levels of THRa1_cyt_gr4.

Means for Survival Time				
THRa1_cyt_gr4	Mean			
	Estimate	Std. Error	95% Confidence Interval	
			Lower Bound	Upper Bound
0	8,492	0,225	8,052	8,933
1	8,686	0,328	8,042	9,33
Overall	8,585	0,187	8,218	8,952

a Estimation is limited to the largest survival time if it is censored.

Overall Comparisons			
	Chi-Square	df	Sig.
Log Rank (Mantel-Cox)	1,253	1	0,263

Test of equality of survival distributions for the different levels of THRa1_cyt_gr4.

Means for Survival Time				
THRa1_cyt_gr4	Mean			
	Estimate	Std. Error	95% Confidence Interval	
			Lower Bound	Upper Bound
0	7,757	0,403	6,967	8,548
1	8,134	0,588	6,981	9,287
Overall	7,92	0,337	7,26	8,58

a Estimation is limited to the largest survival time if it is censored.

Overall Comparisons			
	Chi-Square	df	Sig.
Log Rank (Mantel-Cox)	0,475	1	0,491

Test of equality of survival distributions for the different levels of THRa1_cyt_gr4.

Suppl. Figure S5: Survival Function of nuclear TRα1, nuclear TRα1 is not a significant prognosticator for overall Survival, ten year survival or disease free survival

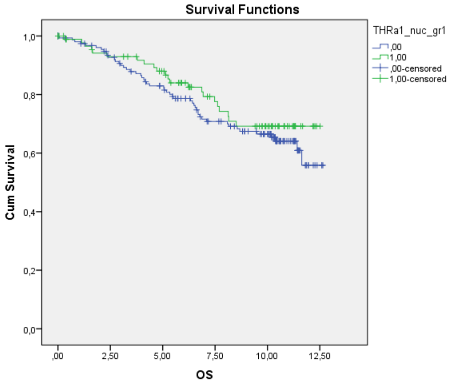

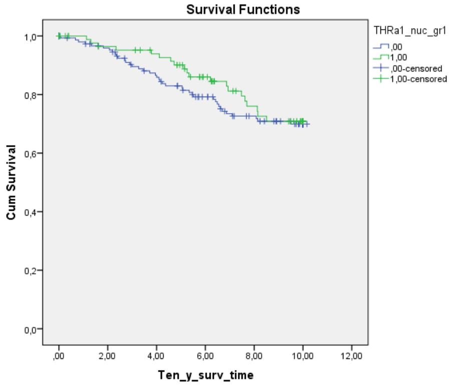

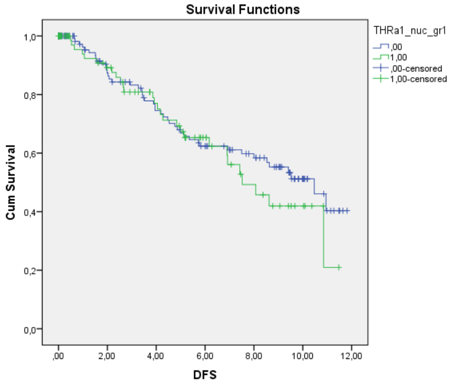

Means for Survival Time				
THRa1_nuc_gr1	Mean			
	Estimate	Std. Error	95% Confidence Interval	
			Lower Bound	Upper Bound
0	9,846	0,335	9,19	10,502
1	10,291	0,414	9,48	11,102
Overall	10,035	0,263	9,52	10,551
a Estimation is limited to the largest survival time if it is censored.				

Overall Comparisons				
	Chi-Square	df	Sig.	
Log Rank (Mantel-Cox)	0,852	1	0,356	
Test of equality of survival distributions for the different levels of THRa1_nuc_gr1.				

Means for Survival Time				
THRa1_nuc_gr1	Mean			
	Estimate	Std. Error	95% Confidence Interval	
			Lower Bound	Upper Bound
0	8,429	0,248	7,942	8,916
1	8,736	0,266	8,214	9,258
Overall	8,585	0,187	8,218	8,952
a Estimation is limited to the largest survival time if it is censored.				

Overall Comparisons				
	Chi-Square	df	Sig.	
Log Rank (Mantel-Cox)	0,321	1	0,571	
Test of equality of survival distributions for the different levels of THRa1_nuc_gr1.				

Means for Survival Time				
THRa1_nuc_gr1	Mean			
	Estimate	Std. Error	95% Confidence Interval	
			Lower Bound	Upper Bound
0	8,062	0,425	7,229	8,895
1	7,422	0,53	6,383	8,46
Overall	7,92	0,337	7,26	8,58
a Estimation is limited to the largest survival time if it is censored.				

Overall Comparisons				
	Chi-Square	df	Sig.	
Log Rank (Mantel-Cox)	0,594	1	0,441	
Test of equality of survival distributions for the different levels of THRa1_nuc_gr1.				
